# Post-Transplant Cyclophosphamide-Based Related Haploidentical Transplantation for Adult Diamond-Blackfan Anemia: Long-Term Survival and Review

**DOI:** 10.14740/jh2220

**Published:** 2026-08-31

**Authors:** Tomohiro Sakakibara, Sumiko Kobayashi, Toshiro Ito, Moriaki Tachibana, Eturo Ito

**Affiliations:** a Department of Hematology, Tokyo Metropolitan Institute for Geriatrics and Gerontology, Tokyo, Japan; b Department of Hematology, National Hospital Organization Matsumoto Medical Center, Matsumoto, Nagano, Japan; c Department of Community Medicine, Hirosaki University Graduate School of Medicine, Hirosaki, Aomori, Japan

**Keywords:** Diamond-Blackfan anemia, Adult, PT-Cy, PBSCT, Haploidentical, Non-malignant, Long-term survival

## Abstract

Diamond-Blackfan anemia (DBA) is a congenital bone marrow failure syndrome (CBMFS) primarily managed with corticosteroids and blood transfusions; however, allogeneic hematopoietic stem cell transplantation (allo-HSCT) remains the sole curative option when these therapies fail or relapse occurs. We report the case of a 21-year-old woman diagnosed with DBA caused by an *RPS19* mutation shortly after birth. Although she responded well to corticosteroid therapy until 18 years of age, discontinuation led to relapse and subsequent transfusion dependence, necessitating allogeneic transplantation. In the absence of a human leukocyte antigen (HLA)-matched donor, she underwent post-transplantation cyclophosphamide (PT-Cy)-based haploidentical peripheral blood stem cell transplantation (haplo-PBSCT) at 21 years of age, utilizing her father as the donor. Myeloablative conditioning was performed using fludarabine, melphalan, and busulfan. For acute graft-versus-host disease (GVHD) prophylaxis, PT-Cy (50 mg/kg on days 3 and 4), tacrolimus, and mycophenolate mofetil were administered, resulting in successful neutrophil engraftment on day 16 post-transplantation. Although the patient developed acute GVHD (grade II), it resolved with corticosteroid therapy. Post-discharge, she was readmitted due to acute gastrointestinal GVHD (grade II), which improved after methylprednisolone administration. She was discharged on day 92 and achieved complete transfusion independence for the past 5 years, making this the first reported case of long-term survival following PT-Cy-based haplo-PBSCT for this condition. Including our patient, there are only four reported cases of allo-HSCT using PT-Cy for adult patients with DBA; notably, all patients survived without developing severe GVHD or infectious complications. Generally, transplant outcomes for DBA worsen with increasing age due to severe GVHD and organ damage secondary to long-term iron overload. Given that DBA is a non-malignant disease, meticulous attention must be paid to prevent unnecessary GVHD and graft failure. In adult patients with DBA, PT-Cy yielded successful engraftment and safety, with no incidence of severe GVHD, leading to long-term survival and minimal complications. These findings suggest that allo-HSCT utilizing PT-Cy remarkably expands donor options for congenital bone marrow failure syndromes. Consequently, this approach is expected to become a promising therapeutic strategy not only for hematologic malignancies but also for non-malignant disorders.

## Introduction

Diamond-Blackfan anemia (DBA) is a congenital bone marrow failure syndrome (CBMFS) caused by genetic mutations, typically represented by *RPS19* mutations, and is characterized by pure red cell aplasia that usually presents in infancy. Standard treatment involves red blood cell (RBC) transfusions and corticosteroid therapy [1, 2]. Approximately 80% of patients initially respond to corticosteroids, and 60–70% eventually become transfusion-independent. However, patients who are refractory to these treatments are candidates for allogeneic hematopoietic stem cell transplantation (allo-HSCT), which remains the only curative option for this disease [1, 2]. In recent years, haploidentical transplantation using post-transplant cyclophosphamide (PT-Cy) has emerged as an alternative donor strategy for hematologic malignancies when matched donors are unavailable [3, 4]. However, its application in DBA, particularly in adult patients with infant-onset disease, remains extremely limited [5–7]. We herein report a case of infant-onset DBA successfully treated with haploidentical peripheral blood stem cell transplantation (PBSCT) with PT-Cy in adulthood, in which well-controlled graft-versus-host disease (GVHD) and sustained erythropoiesis were achieved for more than 5 years.

## Case Report

**Figure 1:**
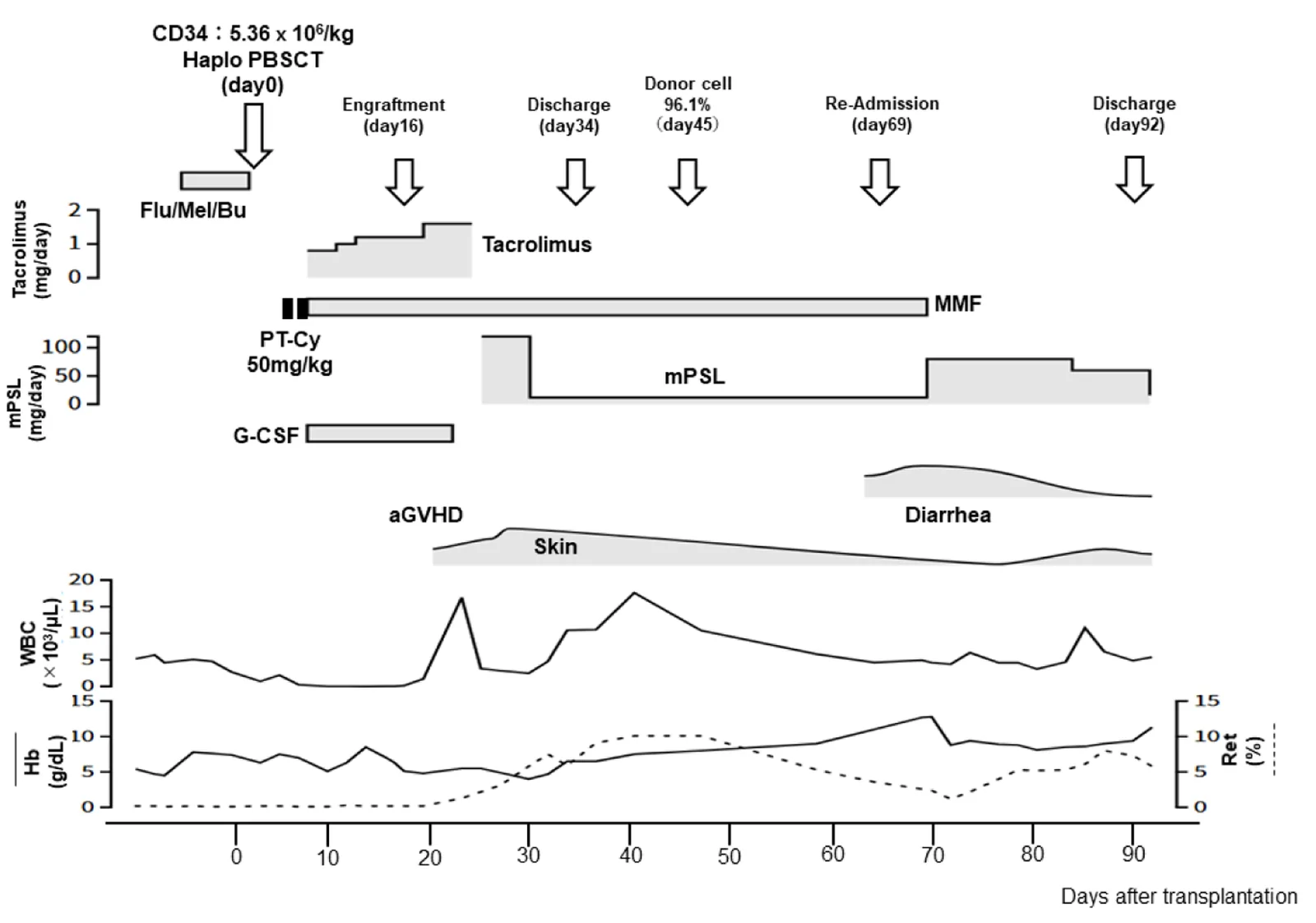
Clinical course of the patient. Adult DBA engrafted after related PT-Cy haplo-PBSCT. Acute skin GVHD developed. However, tacrolimus was discontinued due to malabsorption failing to reach therapeutic levels, and GVHD was managed with methylprednisolone and MMF. GI-GVHD precluded oral medication absorption, requiring management with methylprednisolone monotherapy. After engraftment, the patient achieved complete independence from RBC transfusions. aGVHD: acute graft-versus-host disease; Bu: busulfan (6.4 mg/kg); DBA: Diamond-Blackfan anemia; Flu: fludarabine (150 mg/m^2^); G-CSF: granulocyte-colony stimulating factor; haplo-PBSCT: haploidentical peripheral blood stem cell transplantation; Hb: hemoglobin; Mel: melphalan (140 mg/m^2^); MMF: mycophenolate mofetil; mPSL: methylprednisolone; PT-Cy: posttransplant-cyclophosphamide; ret: reticulocyte count; WBC: white blood cell.

A 26-year-old woman with DBA caused by an *RPS19* mutation (c.356 +1G>A), who was diagnosed at 3 months of age was admitted for allo-HSCT. Following the diagnosis, she achieved remission with prednisolone and cyclosporine therapy, which was discontinued at 18 years of age. However, anemia relapsed at 19 years of age. Although prednisolone was reintroduced, she remained transfusion-dependent. At 20 years of age, she developed steroid-induced psychosis while receiving prednisolone at another hospital, leading to the gradual discontinuation of steroid therapy. Thereafter, she required red blood cell transfusions every week. At 21 years of age, she was referred to our institution for allo-HSCT. On initial evaluation, the laboratory analysis revealed severe anemia with a hemoglobin (Hb) level of 46 g/L and a reticulocyte count of 0.1%. No congenital malformations were observed. Although the patient was registered with the Japan Marrow Donor Program, no suitable donor was identified. Then, her father was chosen as a donor in whom human leukocyte antigen (HLA) was matched and the *RPS19* mutation was absent. Despite the high ferritin level (2,824 ng/mL), echocardiography showed a preserved cardiac function with an ejection fraction of 72.1%, and liver function tests were within the normal range. Although the donor was an HLA haploidentical, GVHD and graft failure were a major concern in the setting of PBSCT from the parent. Therefore, PT-Cy was introduced as a GVHD prophylaxis, replacing the conventional approach. The conditioning regimen consisted of fludarabine (150 mg/m^2^), melphalan (140 mg/m^2^), and busulfan (6.4 mg/kg) as a myeloablative conditioning (MAC) regimen. PT-Cy (50 mg/kg) was administered on days 3 and 4. Tacrolimus (0.02 mg/kg/day) and mycophenolate mofetil (MMF 1,750 mg/day) were initiated on day 5 for GVHD prophylaxis (Fig. 1). Neutrophil engraftment was achieved on day 16, followed by RBC and platelet engraftments on day 20 and day 31, respectively. On day 17, the patient developed grade II acute skin GVHD, and tacrolimus was discontinued because its blood concentration failed to reach the therapeutic range despite a threefold dose escalation during the transplant period, demonstrating a lack of efficacy. Instead, the immunosuppressive regimen was switched to methylprednisolone with MMF as JAK2 inhibitors were unavailable at that time, leading to her discharge on day 34 (Supplementary Material 1, jh.elmerpub.com). The patient achieved full donor chimerism (96.1%) on day 45, as confirmed by sex-mismatched fluorescence *in situ* hybridization (FISH) using bone marrow samples and no longer required transfusion after engraftment. On day 69, the patient was readmitted with grade II acute gastrointestinal-GVHD (GI-GVHD). Given the potential for malabsorption of methylprednisolone and MMF due to watery diarrhea, we stopped MMF and steroids were switched to intravenous administration methylprednisolone monotherapy to ensure rapid efficacy. Her condition improved, leading to her second discharge on day 92. At 5 years after transplantation, the trilineage hematopoiesis remains stable, transfusional iron overload has improved, and the patient has achieved complete social reintegration.

## Discussion

Previous reports have indicated that favorable outcomes in allogeneic HSCT for DBA are associated with age at transplantation of < 10 years, use of an HLA-matched donor, MAC regimen, and bone marrow as the stem cell source [1, 2, 5, 7, 8]. Because older patients are considered to have a longer history of blood transfusions, thereby predisposing them to a higher risk of complications. In addition, matched unrelated donor transplantation has been associated with a higher incidence of chronic GVHD, particularly in children ≥ 10 years of age. Because HLA-matched sibling donors are still considered the preferred donor source for HSCT in patients with DBA [9–11]. Arcuri et al demonstrated the efficacy of myeloablative PBSCT incorporating PT-Cy [12]. The current case diverged significantly from these conventional reports; the patient was 21 years of age and underwent HLA-haploidentical transplantation using peripheral blood stem cells (PBSCs). Nevertheless, the patient has successfully overcome the disease and maintained a stable clinical state for more than 5 years post-transplant. Regarding conditioning regimens, the MAC regimen has traditionally been considered the standard approach for DBA. However, a recent retrospective study involving 27 patients with DBA reported no significant difference in transplantation outcomes between MAC and reduced-intensity conditioning (RIC) regimens, with overall survival rates of 100% and 92.9%, respectively [10, 11]. In stem cell source, for non-malignant diseases, bone marrow is generally preferred over PBSCs because of the lower risk of chronic GVHD [11]. In the present case, however, no suitable matched donor was available. Therefore, haploidentical PBSCT from a related donor was selected. Currently, HLA-haploidentical transplantation with PT-Cy is primarily performed for hematologic malignancies [3, 4]. PT-Cy effectively prevents severe GVHD by eliminating alloreactive T cells while preserving immune reconstitution. However, the optimal dose of cyclophosphamide, particularly in pediatric and young adult populations, has not yet been fully established. Reports from the early 2000s the survival rate for HLA-mismatched donors was extremely low at approximately 23%, and transplantation in adult patients was not recommended at that time in adult DBA [1]. However, the present case demonstrates that the introduction of PT-Cy has enabled haploidentical transplantation (historically associated with high mortality) to be performed safely, even in adult patients with DBA [12–14]. Fuji et al reported a retrospective analysis comparing standard-dose PT-Cy (100 mg/kg) with reduced-dose PT-Cy (80 mg/kg) and found no significant differences in the incidence of acute GVHD (grade II–IV: 29.2% vs. 25.3%; grade III–IV: 7.3% vs. 6.6%) or chronic GVHD [15]. In contrast, Hernandez et al reported on PT-Cy haploidentical transplantation for CBMFS, including DBA. In their study, a cyclophosphamide (Cy) dose of 25 mg/kg led to grade IV acute GVHD in two patients with Fanconi anemia, while two patients with DBA died from engraftment failure [16]. Following these results, the Cy dose was subsequently adjusted to 50 mg/kg. Therefore, it is prudent to reduce the Cy dosage with utmost caution. These findings suggest that optimization of PT-Cy dosing, taking into account long-term cardiotoxicity and fertility, remains an important area for future investigation. To the best of our knowledge, including our case, a total of four cases of allo-HSCT using PT-Cy for adult DBA have been reported [13, 14]. Table 1 summarizes these cases. A common feature among them is that all patients achieved successful engraftment, experienced neither severe GVHD nor significant infections, and survived, with some achieving long-term survival. Although transplantation outcomes for adult DBA are generally known to decline with advancing age, the use of PT-Cy-based transplantation holds promise for improving these outcomes. Future challenges remain regarding whether MAC or RIC is the optimal conditioning regimen, and whether bone marrow or PBSCs remains the preferred graft source, warranting further investigation. Nevertheless, it has become increasingly evident that PT-Cy haploidentical transplantation can ensure safety, guarantee engraftment, and control GVHD, even in the setting of HLA-mismatched transplantation [13, 17, 18]. To date, there have been no previous reports describing successful haploidentical PBSCT with PT-Cy in an adult patient with DBA who had reached adulthood. In DBA, patients ≥ 17 years of age or those undergoing transplantation with multiple HLA mismatches are considered to have a higher risk of transplant-related mortality, including GVHD-related complications [1, 7, 15–17]. In the present case, prompt engraftment was achieved, acute GVHD was well controlled, and stable hematopoiesis was maintained for more than 5 years after PBSCT. Importantly, the patient achieved complete social reintegration. The European Society for Blood and Marrow Transplantation treatment guidelines for aplastic anemia and DBA are formulated based on the presence or absence of iron overload [19]. Although there is currently no mention of the indications for haploidentical transplantation, it might be a potential therapeutic option for the future. In summary, these findings suggest that haploidentical PBSCT with PT-Cy may represent a feasible and effective alternative therapeutic option for patients with DBA, a congenital bone marrow failure syndrome caused by genetic mutations, who lack a suitable donor.

**Table 1:** Reports of Post Transplant Cyclophosphamide Allogeneic Stem Cell Transplantation in Adult Diamond-Blackfan Anemia

Study	Age/sex	Prior therapy	Donor type	HLA	Stem cell source	Conditioning regimen	CD34^+^ cells/kg (/10^6^)	Engraftment Neutrophil RBC/Platelet	GVHD acute chronic	Infection by day100	Outcome	F/U(M)
Leick, et al												
	39/M	Steroid	Brother	Haplo	BM	Flu/Cy/TBI2Gy (+rATG?)	1.54	21	0	None	Alive	63
								N/A / 43	none			
	39/M	Steroid	Cousin	Haplo	BM	Flu/Cy/TBI2Gy (+rATG?)	3.69	21	I (skin stage 1)	None	Alive	12
		Testosterone						N/A / 35	Limited (eye)			
Wirk, et al												
	35/M	Steroid	Unrelated	Match	PBSC	Flu/Cy/rATG/TBI4Gy	5.94	14	0	None	Alive	15
								N/A / 14	None			
Our case	21/F	Steroid	Father	Haplo	PBSC	Flu/Mel/Bu	5.36	16	II (skin, GI)	None	Alive	62
								20 / 31	Limited (eye)			

BM: bone marrow; Bu: busulfan; Cy: cyclophosphamide; F/U: follow-up; Flu: fludarabine; GI: gastrointestinal; GVHD: graft-versus-host disease; HLA: human leukocyte antigen; Mel: melphalan; PBSC: peripheral blood stem cell; rATG: rabbit anti-thymocyte globulin; TBI: total body irradiation.

## Supplementary Material

Suppl 1 Laboratory data during the clinical course of DBA patient.

## Data Availability

The data supporting the findings of this study are available from the corresponding author upon reasonable request.
